# Nontarget Biomolecules Alter Macromolecular Changes Induced by Bactericidal Low–Temperature Plasma

**DOI:** 10.1109/TRPMS.2017.2761405

**Published:** 2017-10-11

**Authors:** A. Privat-Maldonado, Y. Gorbanev, D. O’Connell, R. Vann, V. Chechik, M. W. van der Woude

**Affiliations:** 1 Department of BiologyCentre for Immunology and Infection; 2 Department of PhysicsYork Plasma Institute, University of YorkYorkYO10 5DDU.K.; 3 PLASMANTDepartment of ChemistryUniversity of Antwerp2610AntwerpBelgium; 4 Department of ChemistryUniversity of YorkYorkYO10 5DDU.K.; 5 Centre for Immunology and Infection, Hull York Medical School; 6 Department of BiologyUniversity of YorkYorkYO10 5DDU.K.

**Keywords:** Bacterial inactivation, low-temperature plasma (LTP), plasma-treated media, reactive oxygen and nitrogen species (RONS)

## Abstract

Low-temperature plasmas (LTPs) have a proven bactericidal activity governed by the generated reactive oxygen and nitrogen species (RONS) that target microbial cell components. However, RONS also interact with biomolecules in the environment. Here we assess the impact of these interactions upon exposure of liquid suspensions with variable organic content to an atmospheric-pressure dielectric barrier discharge plasma jet. *Salmonella enterica* serovar Typhimurium viability in the suspension was reduced in the absence [e.g., phosphate buffered saline (PBS)], but not in the presence of (high) organic content [Dulbecco's Modified Eagle's Medium (DMEM), DMEM supplemented with foetal calf serum, and Lysogeny Broth]. The reduced viability of LTP-treated bacteria in PBS correlated to a loss of membrane integrity, whereas double-strand DNA breaks could not be detected in treated single cells. The lack of bactericidal activity in solutions with high organic content correlated with a relative decrease of ^•^OH and O_3_/O_2_(a^1^${\Delta }\text{g}$)/O, and an increase of H_2_O_2_ and $\mathrm{NO}_{2}^{-}$ in the plasma-treated solutions. These results indicate that the redox reactions of LTP-generated RONS with nontarget biomolecules resulted in a RONS composition with reduced bactericidal activity. Therefore, the chemical composition of the bacterial environment should be considered in the development of LTP for antimicrobial treatment, and may affect other biomedical applications as well.

## I. Introduction

The antimicrobial activity of low-temperature plasmas (LTPs) has attracted significant interest in the biomedical field for its possible applications for wound decontamination [1], [2]. The partially ionized gas is an important source of reactive oxygen and nitrogen species (RONS) [2], known to cause oxidative damage to the structure and function of biomolecules in cells [3]–[5]. However, these redox reactions are not restricted to RONS and biomolecules in target cells, as the unspecific action of RONS enables their reaction with biomolecules present in the bacterial environment. This is particularly important for wound decontamination, as biomolecules originating from wound exudates, debris, and host tissue cells could react with LTP-generated RONS. Devices such as the MicroPlaster [6] and the PlasmaDerm [7] are being tested for wound healing and decontamination in patients, and therefore it is important to determine the effect of nontarget biomolecules on LTP efficacy to inform therapeutical application.

LTP has been shown to have a higher bactericidal efficacy when applied in aqueous environments lacking biomolecules compared to those containing biomolecules [8], [9]. Furthermore, LTP treatment leads to damage or changes in a range of biomolecules that each could cause or contribute to the bactericidal activity, including membrane damage and DNA strand breakage [10], [11]. This damage has been documented *in situ*, i.e., in live bacterial cells [12], as well as for purified components [13]. These effects are largely ascribed to RONS, even though the identity and composition of active components vary dependent on the LTP source used. The biological effects of LTP on bacteria in a liquid environment will be a result of gas-phase RONS interacting with the liquid environment and the components within it. This may be of particular relevance when both cells and nontarget biomolecules, (e.g., amino acids, vitamins, and carbohydrates in the direct bacterial environment), are present during LTP application, as is the case in many healthcare settings. Furthermore, biomolecules can alter the physiological state of the bacteria that may facilitate resistance to LTP-induced damage.

In this paper, we aimed to gain insight into the effects of nontarget molecules on the antibacterial action of LTPs. We investigated changes in active components and bactericidal efficacy due to LTP exposure in specific liquid environments. A combination of biological and chemical assays were applied to assess these changes due to specifically exposure to an atmospheric-pressure dielectric barrier discharge (AP-DBD) plasma jet operated at kHz frequency, which we previously used to correlate physical parameters with biological impact [11]. Membrane damage and double stranded DNA breaks in single *Salmonella Typhimurium* cells and overall viability were assessed. To examine the nature of putative damaging species, we analyzed the short- and long-lived species generated in liquid upon LTP exposure. We identified a trend in the type of reactive species produced in the presence or absence of biomolecules and a shift from DNA damage to membrane damage in LTP-treated bacteria. This approach allows correlations to be drawn between types of RONS, bactericidal activity and specific effects on bacterial biomolecules for a specific LTP, and emphasizes the need to consider the complexities of interactions between variable environments, living cells and the identity active LTP components.

## II. Materials and Methods

### A. Plasma Setup and Treatment Conditions

**Fig. 1.**
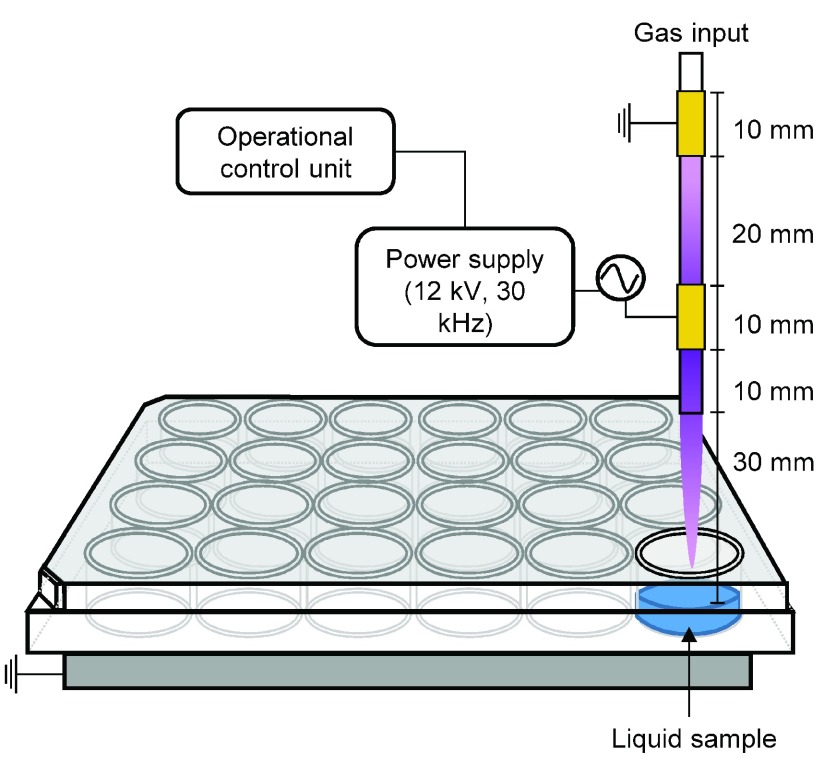
Schematic of the experimental setup.

A parallel field AP-DBD plasma jet described in [11] was used (Fig. 1). Plasma was ignited inside a glass tube (ID 1 mm, OD 6 mm) at 12 kV (peak-to-peak), 30 kHz, with a feed gas flow of 2 slm of helium (99.996%, BOC, U.K.) and 0.5% (v/v) oxygen (99.6%, BOC, U.K.). Samples were treated for 90 s with an ambient humidity ranging from 20%–24%. We have previously demonstrated that this treatment time is sufficient to inhibit growth of this bacterial strain [11]. The distance between the nozzle and the sample was 30 mm. Five liquids with different organic and salts content were used: 1) deionized H2O; 2) phosphate buffered saline (PBS); 3) Dulbecco’s Modified Eagle’s Medium (DMEM, Gibco); 4) DMEM supplemented with 10% (v/v) fetal calf serum (DMEMsupp); and 5) Lysogeny Broth (LB-Miller, Fisher BioReagents).

### B. Bacterial Growth and Sample Preparation

*Salmonella enterica* subsp. *enterica serovar* Typhimurium ST4/74 (*S. Typhimurium*) was grown aerobically at 37 °C in LB. Late logarithmic phase cultures were then diluted in the corresponding liquid (H_2_O, PBS, LB, DMEM, or DMEMsupp) to an optical density at 600 nm of 0.02 (approximately $1.6{\times} 10^{7}$ CFU/mL). These bacterial suspensions were kept on ice until LTP exposure, or plating (for untreated controls), and as briefly as possible (< 15 min). 500 $\mu \text{L}$ of each bacterial suspension were exposed to LTP treatment in 24-well plate as shown in Fig. 1. Sealing films (SealPlate, Excel Scientific) were placed on wells that were not to be exposed to active plasma components. UV does not contribute to the bactericidal activity of this specific LTP [11].

### C. Assessment of Viability and Bactericidal Activity

To assess viability upon LTP treatment, serial dilutions of LTP-treated samples and untreated controls were plated on LB agar plates and the number of colony forming units (CFUs)/mL was determined after overnight incubation at 37 °C.

### D. Live/Dead Bacterial Viability Assay and Cell Sorting

The Live/Dead BacLight Bacterial Viability Assay (Molecular Probes) was used to assess cell viability of *S. Typhimurium* after plasma treatment as a function of the integrity of the cell membrane. The membrane-impermeable nucleic acid dye propidium iodide (PI) stains intracellular nucleic acids only when the cell membrane is damaged. In contrast, the membrane-permeable SYTO9 dye labels the nucleic acid content in all cells. Samples were exposed to LTP treatment, after which they were immediately stained and analyzed. Dye uptake was assessed using the Beckman Coulter CyAn ADP flow cytometer using a 488-nm excitation filter (for both SYTO9 and PI) and 530/40 nm (SYTO9) and 613/20 nm (PI) emission filter. Cells (“events”) were assigned to one of three populations based on the dye uptake: 1) high membrane damage (dead, SYTO9^low^ PI^+^); 2) no membrane damage (live, SYTO9^+^); and 3) cells with moderate membrane damage (intermediate, SYTO9^+^PI^+^) (see Fig. S1 in the supplementary material). A minimum of 10 000 events per sample were acquired. Data were analyzed using Summit version 4.3 software (Beckman Coulter).

For cell sorting, LTP-treated samples were stained as described above and the different identified populations were collected in PBS using the Astrios Eq cell sorter (Beckman Coulter Inc.) with a 488/526- and 561/613-nm excitation/emission filters for SYTO9 and PI, respectively. Four populations were identified for cell sorting: 1) live^low^ (SYTO9^low^, PI^−^); 2) live^high^ (SYTO9^high^ PI^−^); 3) intermediate (SYTO9^+^, PI^+^); and 4) dead cells (SYTO9^low^, PI^+^). Up to 500 000 events per gate were sorted in LB medium and processed to determine viable CFU counts as described in Section II-C. Gating strategy is described in Fig. S1 in the supplementary material.

### E. Double-Strand DNA Breaks in Single Cells Analysis

The presence of double-strand DNA (dsDNA) breaks was assessed in single cells using the DNA damage diffusion (DDD) Assay as previously described [11], [14]. Briefly, bacteria were grown and resuspended in the liquid environments specified above. These suspension were exposed to plasma and immediately embedded in 1% low-melting point agarose for processing. SYBR Gold (Invitrogen) was used to stain the bacterial DNA and the effect of the double stranded DNA breaks in individual cells was visualized by fluorescent microscopy. Visual data were quantified as described [11]. Data was acquired for a minimum of 70 single cells per sample.

### F. Electron Paramagnetic Resonance Spin Trapping

Electron paramagnetic resonance (EPR) and spin trapping was performed in the specified liquids in the absence of bacteria after LTP treatment. The presence of hydroxyl radical (^•^OH), ozone (O_3_)/atomic oxygen (O), singlet oxygen (O_2_($\text{a}^{1}\Delta \text{g}$)), and superoxide radical anion ($\text{O}_{2}^{\cdot -}$) in liquids was assessed by EPR using spin trapping, as described in [15]. Solutions of spin traps 2,2,6,6-tetramethylpiperidine (TEMP), 5,5-dimethyl-1-pyrroline ${N}$-oxide (DMPO), and 5-(diethoxyphosphoryl)-5-methyl-1-pyrroline ${N}$-oxide (DEPMPO) were freshly prepared in the corresponding liquids at 0.1 M concentration prior to use (see Table S1 in the supplementary material). The addition of the singlet delta oxygen scavenger sodium azide (NaN_3_) at a 0.1 M concentration to a solution of TEMP allowed distinguishing between the amount of 2,2,6,6-tertramethylpiperidine 1-oxyl (TEMPO) formed from O_3_/O and that from O_2_($\text{a}^{1}\Delta \text{g}$)) [16]. EPR spectra simulations were performed on NIH P.E.S.T. WinSIM software version 0.96. Simulated spectra were double-integrated with SpectrumViewer Plus version 2.6.3. For simulations, the hyperfine coupling constants were obtained from [17]. The pH of the solutions was monitored using the Hydrion Insta-check 0–13 pH test paper (Sigma). The calibration of the EPR signal (double integral intensity as a function of a radical concentration) was done using a stable radical TEMPO (Sigma Aldrich) as shown in Fig. S2 in the supplementary material. Representative EPR spectra of the spin trap adducts are shown in Fig. S3 in the supplementary material.

### G. Colorimetric Assays

Colorimetric assays were used to quantify H_2_O_2_ and nitrite (NO_2_^−^). These were carried out after applying LTP to the specified liquid in the absence of bacteria. Specifically, the potassium titanium(IV) oxalate method was used to measure H_2_O_2_ as described in [15]. Immediately after plasma treatment, $500~\mu \text{L}$ of the titanium(IV) solution were mixed with $300~\mu \text{L}$ of the plasma-treated liquid and immediately analyzed by UV-V is absorption. H_2_O_2_ concentrations were determined from the absorbance maxima at 400 nm. The Griess assay (Sigma Aldrich) was used to detect nitrite (NO_2_^−^). After treatment, $400~\mu \text{L}$ of each sample were mixed with $400~\mu \text{L}$ Griess reagent and incubated for 5 min in the dark at room temperature. NO_2_^−^ concentrations were determined from the absorbance maxima at 526 nm. Absorbance values were converted to concentrations using calibration curves obtained with H_2_O_2_ (30 wt%, Fluka) and NaNO_2_ ($\geq 97.0$%, Sigma Aldrich) (Fig. S4 in the supplementary material). Concentrations quoted are after initially correcting for background absorbance and finally for liquid evaporation that occurred during LTP treatment.

## III. Results and Discussion

### A. Bactericidal Activity is Reduced in Solutions With High Organic Content

**Fig. 2.**
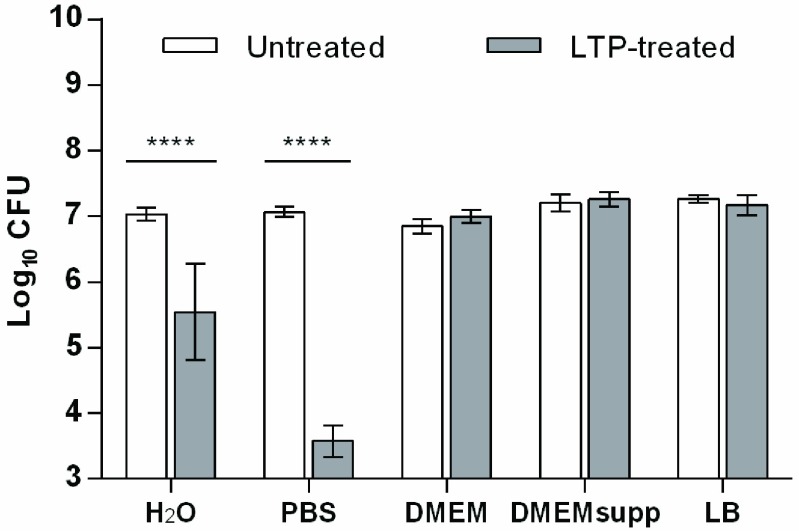
Effect of external organic content on bacterial viability. CFU Log_10_ reduction of *S. Typhimurium* in the five solutions exposed to plasma. Data presented as mean ± S.D. ****: ${P} <0.0001$.

We investigated the impact of varying organic and inorganic content on the bactericidal effect of LTP in a liquid environment, and addressed key mechanisms of action. This was assessed using a gram-negative model bacterium, *S. Typhimurium*. The bactericidal effect, or “inactivation” is defined here as the condition under which single bacteria are unable to proliferate to generate visible colonies on agar plates (CFUs). We observed a 3.5-log reduction in viable bacteria exposed to LTP in PBS (99.9%–99.99% inactivation) and 1.5-log reduction in H_2_O (90%–99% inactivation) ($P<0.0001$; Fig. 2). We previously showed that UV does not contribute to the bactericidal activity of the LTP used here [11]. Therefore, although *S. Typhimurium* has redundant pathways for protection from oxidative stress [18], the oxidative damage to cells by LTP treatment in PBS was lethal. In contrast, using the same LTP parameters and exposure time, no reduction in viability was observed when *S. Typhimurium* was treated in liquids with high organic content (DMEM, DMEMsupp, and LB). These results correlate with previous findings on the gram-positive bacteria *Staphylococcus aureus* exposed to a spark-based nonthermal plasma [8]. The discovered differences in the bactericidal of activity of LTP in the media studied was a result of the different amounts and nature of RONS available (see below).

**Fig. 3.**
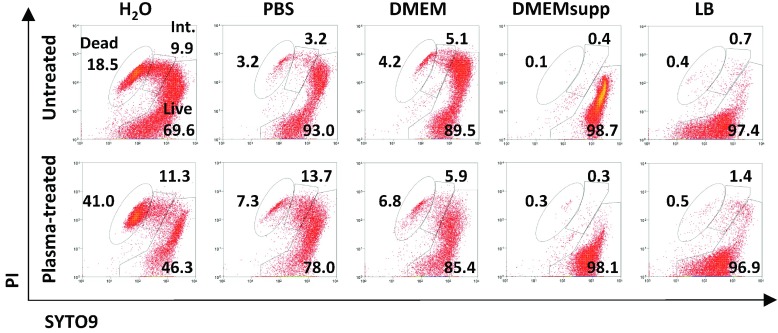
Representative PI/SYTO9 dot plots of untreated and LTP-treated *S. Typhimurium* in the five liquids tested. The gating areas represent dead, intermediate (int.), and live populations.

Membrane integrity is vital for bacterial survival, and the cell membrane is the first barrier that protects cells from external oxidative stress [19]. LTP treatment can cause etching [20], lipid peroxidation [21], and overall cell membrane damage [22], which can contribute to the LTP bactericidal activity. We assessed membrane integrity by uptake of the nucleic acid dye PI, to which cells with intact membranes are impermeable (see methods). Fig. 3 shows representative dot plots of the flow cytometry data showing signals for PI and SYTO9 dye, which stains nucleic acids in all cells, in individual cells; cumulative data are presented in Table S2 in the supplementary material. LTP exposure reduced the number of bacteria with intact cell membrane (“live”; SYTO9^+^ PI^−^) in PBS ($P<0.05$), DMEM ($P<0.01$), and DMEMsupp ($P<0.001$) (Table S2 in the supplementary material). For bacteria treated in PBS, the identified decrease in membrane integrity correlated with a loss in viability as determined by CFU (Fig. 2). The difference in relative percentages could be due to the differences in the method identifying “viable” bacterial cells and the time points at which viability is assessed. CFU counts reflect the ability to grow and divide, and were assessed after 18 h of incubation, whereas flow cytometry counts were assessed immediately after treatment and only reflects membrane damage. Thus the CFU assays incorporate a period that allows for active recovery. Furthermore, membrane components may be damaged such that PI uptake is not increased but nevertheless cause cell death, for example a change in cell membrane fluidity [23]. In the population of bacteria treated in DMEM, there was a small but significant increase in the percentage of cells with high cell membrane damage (“dead”; SYTO9^low^ PI^+^; $P<0.05$) (Fig. 3 and Table S2 in the supplementary material). However, over 99% of cells retained viability after treatment in this medium, as determined by CFU (Fig. 2), and thus the impact of this damage on bacterial viability is not apparent.

**Fig. 4.**
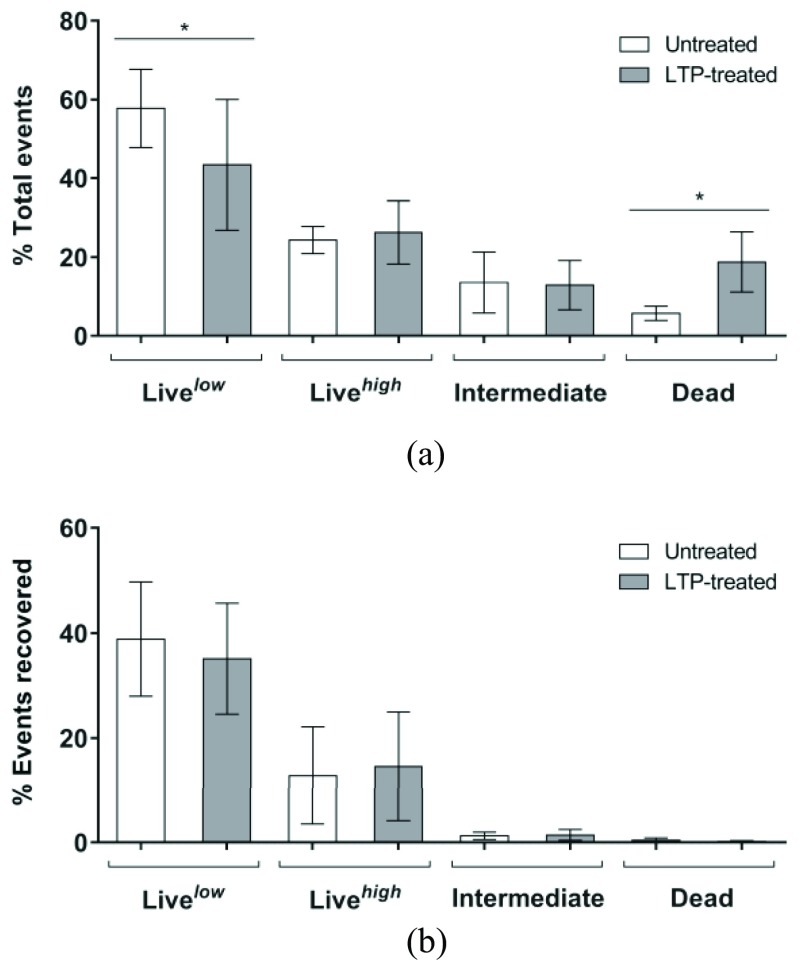
Cell sorting of live/dead stained *S. Typhimurium* suspensions in PBS. (a) Cell sorting identified four populations: 1) live^low^; 2) live^high^; 3) intermediate; and 4) dead. (b) Viability of 500 000 events from each population of *S. Typhimurium* in PBS. Data presented as mean ± S.D. *: $P<0.05$.

More than 99% of all cells treated in PBS lost viability as determined by CFU, yet subpopulations differed in level of membrane damage as determined by SYTO9/PI staining profiles. Analysis of the *live* population data using a slightly different set of filters with the cell sorter allowed a further distinction into two subpopulations: 1) live^low^ (no membrane damage and low nucleic acid content) and 2) live^high^ (no membrane damage and high nucleic acid content). To assess the viability and potential to recover from membrane damage in LTP-treated bacteria in PBS suspensions, we sorted populations according to SYTO9/PI staining profiles and determined viability. This was expressed as the percentage of cells (*events*) in a subpopulation that gave rise to a colony. LTP treatment reduced the percentage of live^low^ cells and increased the percentage of membrane damaged, i.e., *dead* cells ($P<0.05$; Fig. 4(a); Table S2 in the supplementary material), whereas the live^high^ and intermediate percentages remained similar. An increase in the percentage of the intermediate population was not observed in our cell sorting experiment (Fig. 4) as initially observed in the flow cytometric assay (Fig. 3 and Table S2 in the supplementary material). This difference could be because a live^high^ subpopulation may not have been distinct from the intermediate population in the method used for Fig. 3. The highest percentages of cells generating CFU (i.e., viability) were obtained in the live^low^ and live^high^ populations, as expected [Fig. 4(b)], while in the intermediate and dead subpopulations (SYTO9^+^PI^+^) only a low percentage of cells generated colonies, irrespective of LTP treatment. Cells therefore appear to be able to recover to a very low degree from membrane damage, as identified in the intermediate population. However, as this level of damage is not increased by LTP, it represents intrinsically occurring membrane permeability.

Overall, LTP treatment of cells in PBS did not alter the recovery profiles of all four studied populations, but in contrast resulted in a shift of cells from the PI/SYTO9 subpopulation live^low^ to dead. This indicates the live^low^ population, with a lower overall nucleic acid content, is more susceptible to LTP induced bactericidal activity. The assay does not distinguish between RNA or DNA, but this may indicate that cells with lower genome content as occurs immediately after cell division, are less robust than cells with higher DNA content. Whether in these cells DNA damage is more lethal, or whether it is related to associated features like cell size and surface area, remains to be determined.

### B. LTP Induced Low Levels of Double-Strand DNA Breaks in S. Typhimurium Suspensions

**Fig. 5.**
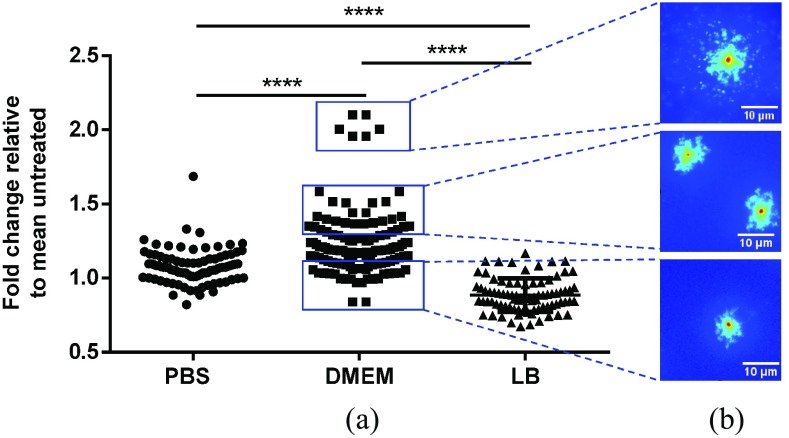
dsDNA breaks in LTP-treated *S. Typhimurium* suspensions. *S. Typhimurium* suspended in PBS, DMEM, and LB were treated with the AP-DBD plasma jet for 90 s and analyzed with the DDD assay. (a) Fold change in bacterial DNA diffusion in plasma-treated bacteria relative to the mean of untreated bacteria. Each dot represents a single cell; horizontal bars: mean values ± S.D.; ****: $P<0.0001$. (b) Representative single cells showing the level of DNA fragmentation observed in plasma-treated *S. Typhimurium* in DMEM suspension. Scale bar 10 mm.

DNA breaks in bacterial genomes are lethal if they cannot be repaired [24], and LTP can induce DNA strand breaks [11]. Using the DDD assay, we assessed the integrity of *S. Typhimurium* genomic DNA in single cells after plasma treatment in PBS, LB, and DMEM (Fig. 5). LTP treatment of bacteria in LB and PBS suspensions did not lead to detectable levels of dsDNA breaks whereas in DMEM suspensions a low but significant increase in cells with dsDNA breaks occurred as a result of LTP treatment (Fig. 5). This level of damage is much lower as we previously determined for cells exposed to the same LTP source on a solid surface [11]. It is not certain why the minor increase in damage is observed specifically and only in DMEM. One possible explanation could be the formation of H_2_O_2_ in DMEM by the oxidation of amino acids (such as cysteine, histidine, methionine, phenylalanine, and tryptophan) by O_3_ [25]. In addition, iron ions in complex media could facilitate the formation of ^•^OH (*via* Haber-Weiss and Fenton reactions) which contributes to DNA damage [26]. Regardless, in the parallel experiment (Fig. 2), no loss of viability occurred in cells in DMEM exposed to LTP, suggesting that the observed, low level of DNA damage is not leading to cell death in a detectable fraction of the population.

The data show that LTP application to cells in liquid suspension did not induce high levels of dsDNA breaks, even in PBS where cell death was apparent. It is possible that the levels of dsDNA damage inflicted to cells were below the sensitivity of the DDD assay (as a single dsDNA break cannot be identified), or other type of lethal DNA damage such as single-strand DNA breaks and nucleobase oxidation took place. Alternatively, DNA damage may not be a significant contributing factor to bacterial cell death in these conditions.

Taken together, our data suggests that not all DNA damage is preceded by (detectable) damage to the cell membrane. It is possible that under the conditions used here, either damage to the cell membrane was not detectable, or did not occur and after uptake of an initiation component, the DNA damage was effected by reactions that occurred within the cell.

### C. Nutrients in Solution During LTP Treatment Altered the Levels of RNOS Available

The bactericidal activity of LTP is governed by the RONS generated by plasma and delivered to the biological substrate. We assessed the presence of some of the biologically relevant RONS created by direct plasma treatment [2], [16], specifically ^•^OH, H_2_O_2_, O_3_/O, O_2_($\text{a}^{1}\Delta \text{g}$), $\text{O}_{2}^{\bullet ^{-}}$, and NO_2_^−^ in the specified liquids in the absence of bacteria. The H_2_O_2_ and NO_2_^−^ concentrations were assessed colorimetrically. The amounts of the highly reactive and very short-lived free radicals were assessed by EPR using the spin traps TEMP, DMPO, and DEPMPO. The spin traps react with the studied radicals, forming more persistent radical adducts which can be detected by EPR.

**Fig. 6.**
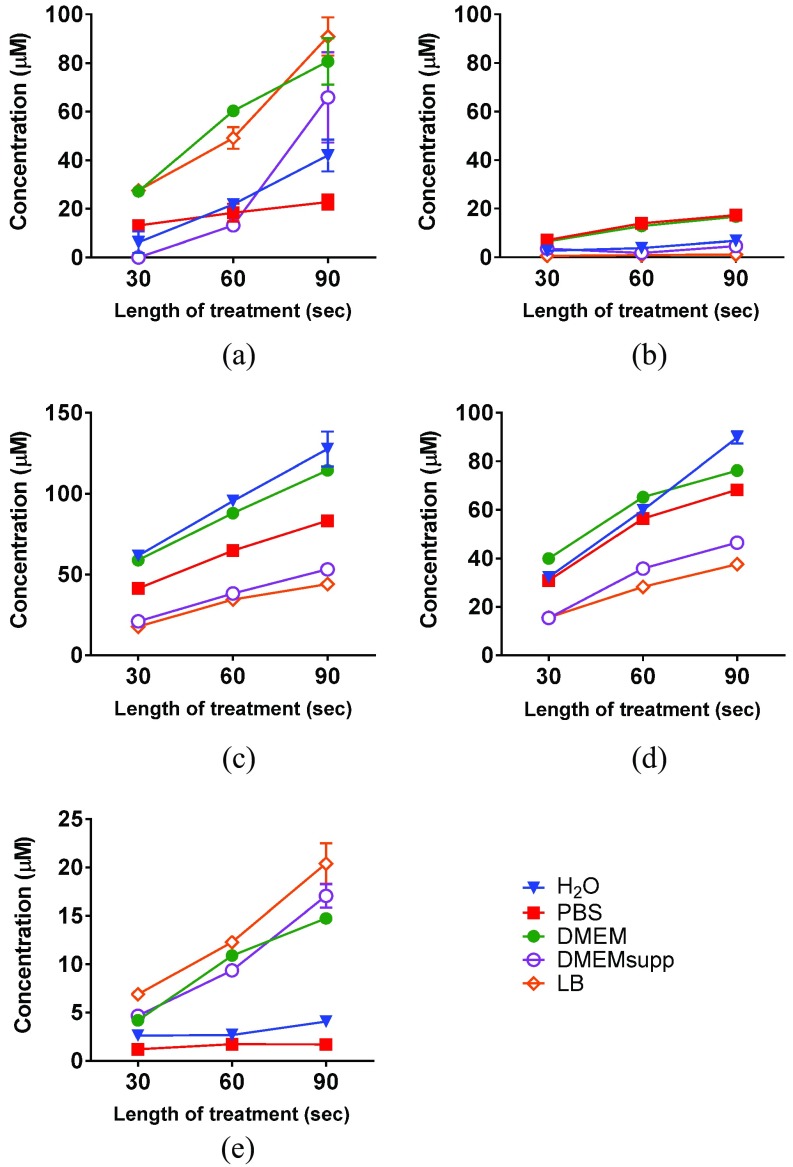
Detection of RNOS in plasma-treated liquids. (a) Colorimetric assay for the detection of H_2_O_2_ by reaction with titanium(IV). Spin-trapped radical adducts of (b) DMPO-OH adduct, (c) TEMPO without added NaN_3_, and (d) TEMPO with added NaN_3_. Horizontal bars: mean ± S.D. (e) Azo dye formed by NO_2_^−^, assessed by Griess assay. Single measurements for 30- and 60-s treatments and mean values for 90-s treatment ($n = 2$). Length of treatment expressed in seconds.

We acknowledge that other biologically relevant reactive species not measured in this paper may be present in the reactive systems (e.g., peroxynitrite anion [27]). In all LTP-treated samples, longer treatment time resulted in an increase in the concentration of the detected spin trap adducts, azo dye and peroxide compounds used in this paper (see Section II), which was caused by an increase of the amount of the relevant active species (Fig. 6).

The highest levels of H_2_O_2_ occurred in LTP-treated DMEM, DMEMsupp, and LB [Fig. 6(a)]. A similar increase of H_2_O_2_ in plasma-treated DMEM was observed in [26]. The assay with Ti(IV) oxalate was specific to H_2_O_2_, as the addition of catalase abrogated the titanium(IV)-peroxide complex detected [Fig. S5(a) in the supplementary material]. After the plasma exposure, the amount of H_2_O_2_ decreased over time to a higher extent in solutions without organic content [Fig. S5(b) in the supplementary material]. The decay was possibly due to the reaction of H_2_O_2_ with NO_2_^−^ [28], or H_2_O_2_ with solubilized O_3_ [29]. Since most of the H_2_O_2_ found in LTP-treated water originates in the gas phase [15], the increase of H_2_O_2_ in LTP-treated solutions with high organic content could possibly be explained by the reaction of O_3_ with unsaturated aromatic amino acids and aliphatic compounds [30] to form peroxides and H_2_O_2_. This is in agreement with the low levels of O/O_3_ found in DMEM, DMEMsupp and LB compared to H_2_O and PBS (Fig. 6). Thus, our results support the hypothesis that the presence of organic molecules during plasma treatment enhances the formation of H_2_O_2_.

In contrast with H_2_O_2_ concentration, solutions with high organic content yielded the lowest levels of the nitroxide radicals DMPO-OH, TEMPO from O_2_($\text{a}^{1}\Delta \text{g}$), and TEMPO from O_3_/O [Fig. 6(b)–(d)]. Interestingly, the concentration of the DMPO-OH adduct was higher in PBS and DMEM than in water [Fig. 6(b)]. This is unexpected since both chloride and bicarbonate anions (components of the respective media) were suggested to react with hydroxyl radical, reducing its amount [31], [32]. The presence of other inorganic salts in DMEM (e.g., iron ions) and PBS could increase the amount of the available ^•^OH. Another explanation could be different rates of decay of the radical adduct in the explored solutions (minor pH variations, media components, etc.). DMPO-OH was also measured in different concentrations of PBS ($0.5{\times}$–$4{\times}$), and the highest levels occurred at higher concentrations (Fig. S6 in the supplementary material). This may be due to the increased ionic strength and hence conductivity that may favor the formation of ^•^OH [33]. At the same time, the impact of the scavenging ability of chloride was apparently less significant, as the concentration of DMPO-OH in PBS remained high. Low amounts of DMPO-OH in DMEMsupp were seen possibly because any increase in ^•^OH formation was counterbalanced by the consumption of ^•^OH with biomolecules. The spin traps DEPMPO (for the detection of $\text{O}_{2}^{\bullet ^{-}}$) and DMPO (for ^•^OH radicals) both formed only ^•^OH radical adducts. This indicates the absence of detectable amounts of $\text{O}_{2}^{\bullet ^{-}}$ in LTP-treated solutions (Figs. S3 and S7 in the supplementary material).

Similarly to H_2_O_2_, higher levels of NO_2_^−^ were found in solutions with high organic content [DMEM, DMEMsupp, and LB; Fig. 6(e)]. We tentatively attribute the increased levels of NO_2_^−^ found in these media to the consumption of oxidative species [e.g., O_3_ and O; Fig. 6(d)] required to transform NO_2_^−^ *via* further oxidation into NO_3_^−^. The lower amount of NO_2_^−^ in water could be due to the reaction of nitrites with H_2_O_2_, or ^•^NO (the precursor of NO_2_^−^) with $\text{O}_{2}^{\bullet ^{-}}$ [27], [30]. LTP-treated water undergoes acidification [34], [35], favoring these reactions. However, in this paper the changes in the pH values of all LTP-treated solutions did not exceed 0.5 (data not shown).

Thus, the data suggest that different factors, e.g., ionic strength of the LTP-treated solutions may affect the availability of RONS as in the case of different concentrations of PBS (see above). However, the more prominent factor is the presence of organic molecules (which consume oxidative species).

We thus identified a trend showing that the presence of organic content in plasma-treated solutions affects the final concentrations of reactive species, and that these are determined by consumption of short-lived reactive oxygen species and formation of long-lived reactive species. This can only be identified as a trend, since the absolute values of RONS cannot be assessed due to the semiquantitative nature of some of the analyses (e.g., amount of DMPO-OH does not correspond to the total amount of the •OH radical in the liquid). The data further indicate that organic molecules interact with RONS, and suggest that among the reactive species assessed in this paper, ROS play a more relevant role than RNS in bacterial inactivation, especially the O_3_ and •OH that was identified in PBS. Other biologically relevant species such as peroxynitrite not analyzed here could participate in the induction of oxidative damage to bacteria.

Nosenko *et al.* [36] observed that shorter treatments with a plasma torch were required to achieve 100% elimination of *Escherichia coli* in PBS compared to LB suspensions. The effect was attributed to the synergistic activity of ^•^NO and H_2_O_2_. In our case, the findings suggest that the reduced bactericidal action in LTP-treated LB could be a consequence of the loss of reactive oxygen species (e.g., O_3_). The interaction of these species with LB components may lead to the formation of secondary ROS (H_2_O_2_ [30]) at concentrations that have no apparent effect on the viability of bacterial cells.

Our results suggest that the local environment influences the mechanisms of action used by LTPs to induce oxidative damage to targeted cells. A similar phenomenon has been reported for more traditional antiseptics, for example chlorhexidine and silver compounds [37], [38], as their antimicrobial activity is greatly affected by the presence of exogenous organic matter. Whereas membrane damage was observed in LTP-treated bacteria in PBS, DMEM, and DMEMsupp (Fig. 4), bacterial elimination was abrogated in suspensions with high organic content (Fig. 2). In contrast, it is possible that the presence of external organic content such as aromatic amino acids and vitamins (in DMEM) but not peptides (as in LB) [39], [40] during LTP treatment favored the induction of nonlethal dsDNA breaks in *S. Typhimurium* (Fig. 5). Although it is important to understand the interaction of the plasma-induced RONS with the individual components of the environment, here we evaluated the combined effect of all agents present. Taking these data together, this paper suggests that the interaction of plasma-generated RONS with nontarget organic matter is likely responsible for the decrease of the bactericidal activity of LTPs. Our approach of combining a range of assays proved valuable to assess the biological and chemical effects of LTP treatment within a specific set of conditions—LTP source, environment, and biological target.

## IV. Conclusion

The complex interactions between RONS delivered by LTP and organic molecules in biological systems generate additional reactive species and potentially cytotoxic by-products (e.g., the increased amounts of H_2_O_2_ and NO_2_^−^ in LB and DMEMsupp). However, their presence does not necessarily lead to a significant bacterial inactivation, as we show here and indeed can decrease the overall bactericidal effect. The bactericidal effect of LTP-treated *S. Typhimurium* is likely a consequence of a sum of different interactions at the molecular level caused by the synergistic effect of RONS. This damage cannot be ascribed only to one or more specific species measured but to the various reactions with the short- and long-lived species.

Many *in vitro* studies of biomedical plasmas with a range of applications for both eukaryotic and prokaryotic cells use variable conditions, including the presence of organic molecules [12], [27], [41]–[43]. As we described, these organic nontarget components are active participants in the overall process, and their reactions must be considered for the correct interpretation of results. The approach used in this paper aimed to provide different scenarios where different amounts of salts and nutrients (amino acids, peptides, carbohydrates, and vitamins) were present. This is relevant in the context of topical wound treatments with LTPs, where the complex composition of the wound exudate could interfere with the efficacy of the treatment. Our approach demonstrates the effect of nontarget molecules during LTP-treatment of bacteria. Taking previously published findings together with the results described here, there is significant evidence of an adverse effect of external organic molecules on the bactericidal action of LTPs, a key point that should be considered when developing strategies for individual or combined antibacterial LTP treatments in the clinical setting.
